# Deep Learning-Aided Inertial/Visual/LiDAR Integration for GNSS-Challenging Environments

**DOI:** 10.3390/s23136019

**Published:** 2023-06-29

**Authors:** Nader Abdelaziz, Ahmed El-Rabbany

**Affiliations:** 1 Department of Civil Engineering, Toronto Metropolitan University, Toronto, ON M5B 2K3, Canada; rabbany@torontomu.ca; 2 Department of Civil Engineering, Tanta University, Tanta 31527, Egypt

**Keywords:** INS/LIMO, INS/LIMO/LiDAR, integrated navigation system, GNSS-denied environments

## Abstract

This research develops an integrated navigation system, which fuses the measurements of the inertial measurement unit (IMU), LiDAR, and monocular camera using an extended Kalman filter (EKF) to provide accurate positioning during prolonged GNSS signal outages. The system features the use of an integrated INS/monocular visual simultaneous localization and mapping (SLAM) navigation system that takes advantage of LiDAR depth measurements to correct the scale ambiguity that results from monocular visual odometry. The proposed system was tested using two datasets, namely, the KITTI and the Leddar PixSet, which cover a wide range of driving environments. The system yielded an average reduction in the root-mean-square error (RMSE) of about 80% and 92% in the horizontal and upward directions, respectively. The proposed system was compared with an INS/monocular visual SLAM/LiDAR SLAM integration and to some state-of-the-art SLAM algorithms.

## 1. Introduction

Accurate positioning is one of the key challenges in the field of vehicular navigation. A robust navigation system should work under all driving environments (i.e., urban areas, rural roads, high-traffic, or low-traffic) and conditions (i.e., dry, wet, day, or night). In addition, the concept of sensor redundancy is crucial when designing a navigation system. A navigation system should be equipped with several integrated onboard sensors so that if one sensor malfunctions for any reason, the other sensors will allow the system to operate safely and properly [1,2,3]. The global navigation satellite system (GNSS)/inertial navigation system (INS) integration has been well-studied and adopted in a large number of studies [4,5,6,7,8,9,10,11]. Typically, the measurements of both the GNSS and the inertial measurement unit (IMU) are fused using a Kalman filter [12] that features the use of several integration methods between the GNSS and the IMU [13] (i.e., loosely coupled and tightly coupled). In a loosely-coupled integration, for instance, the INS estimates the pose of the vehicle via IMU mechanization while receiving updates from the GNSS at a slower frequency, which minimizes the significant drift of the IMU. The main problem with such navigation systems is that they solely rely on the INS in cases of prolonged GNSS signal outages. This makes the whole system vulnerable to degraded positioning accuracy as a result of the INS solution drift, especially when using a low-cost micro-electro-mechanical system (MMEMS) IMU. Consequently, the need for more onboard sensors is crucial to help maintain a navigation system’s accuracy during a GNSS signal outage.

Light detection and ranging (LiDAR) sensors and cameras have been heavily under investigation to be used for both indoor and outdoor navigation using LiDAR and visual simultaneous localization and mapping (SLAM) techniques. SLAM algorithms are designed to create a map of the surrounding environment while concurrently monitoring the location of the sensor, which is achieved by a LiDAR sensor or a camera [14].

There are a number of state-of-the-art LiDAR SLAM algorithms. One of the key leading algorithms proposed in 2014 is LiDAR odometry and mapping (LOAM) [15]. Back then, LOAM was the leading algorithm on the KITTI odometry benchmark [16]. Subsequently, many variations of the LOAM were developed, such as A-LOAM and Kitware [17,18,19,20]. These variations improved the computational performance of the original LOAM algorithm. It is worth mentioning that one key strength of LiDAR SLAM is that its performance is not affected by illumination conditions because LiDAR is an active sensor.

Similar to the LiDAR SLAM developed algorithms, many visual SLAM (V-SLAM) algorithms have been proposed, as collected and presented in [21]. V-SLAM is a type of SLAM algorithm that utilizes visual cues, such as features or landmarks, to create a map of the environment and determine the location of the sensor. V-SLAM typically uses a camera as the primary sensor and processes images to identify features in the environment. By analyzing the movement of these features between successive images, V-SLAM can estimate the sensor’s location and construct a map of the surrounding area. V-SLAM can be implemented using several types of cameras, such as monocular cameras [22,23,24,25,26] (trajectory is retrieved up to a scale factor) and stereo cameras [27,28,29] (scale factor is corrected). The main advantage of V-SLAM is that it captures significantly more details about the environment in comparison to the use of LiDAR. However, V-SLAM is significantly affected by illumination conditions, unlike LiDAR. In light of the mentioned advantages of both LiDAR sensors and cameras, it is important to mention that the optimal scenario would be to integrate the use of both sensors (i.e., the camera and LiDAR) in order to obtain rich details about the environment while mitigating any adverse effects of the illumination variation.

There are a number of studies that integrated the INS into a LiDAR sensor and/or a camera. For example, in [30], we proposed an integrated INS/LiDAR SLAM navigation system for outdoor environments. The developed integrated system showed superior performance in comparison to the sole use of the INS in all case studies on urban and rural driving environments. Similarly, in [31], the integration between the INS and LiDAR SLAM remedies a malfunctioning GNSS in an unmanned aerial vehicle (UAV) mapping system. The developed GNSS/INS/LiDAR SLAM system was able to fill in the GNSS gaps and overcome the GNSS unexpected outages issue. In [32], an EKF was used to combine a three-dimensional reduced-inertial-sensor system (3D-RISS) with GNSS and LiDAR odometry (LO). The LO provided updates to the position and azimuth of the 3D-RISS. The system was tested in various driving situations in Toronto and Kingston, Canada, and the integration yielded a 64% decrease in positioning errors compared with the sole use of the INS solution. GNSS/IMU/camera integration is exemplified in [33]. The study presented a navigation method that integrates data from a monocular camera, IMU, and GNSS to navigate ground vehicles in environments where GNSS signals are weak or unreliable. The proposed method uses a Kalman filter to estimate the vehicle’s position and orientation by fusing the different sensor data. Experimental results show that the proposed approach outperforms a baseline method that only uses GNSS data in challenging GNSS environments, reducing the position error by up to 74% in some scenarios. The method also demonstrates good robustness against signal outages and multipath interferences. In [34], a loosely-coupled integration between the INS, LiDAR SLAM, and visual SLAM was accomplished using an EKF. The developed integrated system was tested during a full GNSS signal outage using drives of the KITTI dataset [35,36], where the system yielded accurate positioning results in comparison to the INS.

In recent years, the use of deep learning for localization and navigation has been trending. In [37], a monocular camera was integrated into a LiDAR sensor to correct the scale ambiguity of visual odometry with the help of deep learning, which was used in the form of imagery semantic segmentation. In [38], a novel method for LO that utilized deep learning-based feature points and a two-step pose estimation approach was proposed. The first step involved extracting feature points from the LiDAR point cloud data using a convolutional neural network (CNN), which was trained to identify distinctive and robust features. The second step involved estimating the pose of the LiDAR sensor by minimizing the difference between the predicted feature points and the corresponding feature points in the current frame. The proposed method was evaluated using the KITTI dataset and compared with several state-of-the-art LO methods. The experimental results showed that the proposed method achieves higher accuracy and robustness, particularly in challenging scenarios such as fast motion and occlusion. Moreover, the proposed method has a lower computational cost than some of the existing methods, making it more practical for real-time applications. A review of the use of deep learning in localization is presented in [39].

In this research, we propose an INS/monocular visual SLAM integrated system, where a loosely-coupled integration is implemented between the INS solution and the LIMO algorithm (monocular visual SLAM) using an EKF, with the aid of deep learning for imagery semantic segmentation. The developed model is then tested using the raw KITTI dataset in both urban and residential driving environments. It is worth mentioning that the KITTI dataset was considered in our research as a source of low to moderate traffic environment. Therefore, to expand the testing of our model in high-traffic environments, we used the Leddar PixSet dataset.

Subsequently, we extended our work in [30], which developed an INS/LiDAR SLAM integrated navigation system, to include the LIMO algorithm as a part of the navigation system. As a result, an integrated INS/LiDAR/LIMO navigation system was developed for two reasons. The first reason was to investigate the inclusion of the LiDAR sensor as an additional onboard sensor to the IMU and the monocular camera. Secondly, we tested our proposed INS/LIMO navigation system against the INS/LiDAR/LIMO and certain state-of-the-art navigation algorithms.

## 2. Navigation System Architecture

### 2.1. LiDAR SLAM

The LiDAR SLAM used in this research is the Kitware SLAM [17], which is an updated version of the original LOAM algorithm [15]. A detailed description of the Kitware SLAM and the different enhancements it provides in comparison to LOAM are presented in our work in [30].

### 2.2. LiDAR/Monocular Visual SLAM Integration

The LIMO [37,40] algorithm was adopted in our research to integrate the LiDAR sensor into the monocular camera. The LIMO algorithm can be divided into two main stages, namely, monocular visual odometry and scale correction. The first stage involves the use of a conventional visual odometry pipeline, starting from the feature extraction and matching to frame-to-frame motion estimation. The second stage is the key to correcting the scale ambiguity of the resulting trajectory from the first stage, which is accomplished using the LiDAR sensor. Before projecting the LiDAR points into the camera frame and the image plane, all images are semantically segmented to identify the moving objects.

Semantic segmentation refers to the task of assigning each pixel in an image to a specific object class. This is a challenging computer vision task that has many practical applications, such as autonomous driving. One of the leading state-of-the-art models is the original NVIDIA semantic segmentation model [41]. The model was designed to be highly accurate and efficient, which led to the processing of large volumes of imagery data in real time. The model uses an encoder–decoder architecture with skip connections. The encoder consists of a series of convolutional layers, followed by batch normalization and ReLU activation. The convolutional layers use small filters (3 × 3 or 1 × 1) and are designed to reduce the spatial dimensionality of the image while increasing the number of feature channels. The decoder consists of a series of upsampling layers, followed by convolutional layers with batch normalization and ReLU activation. The upsampling layers use bilinear interpolation to restore the spatial resolution of the image. The skip connections are implemented by concatenating the feature maps from the encoder with the upsampled feature maps from the decoder. The output layer of the model is a convolutional layer with softmax activation. This layer assigns a probability distribution over the object classes to each pixel in the image. The class with the highest probability is then assigned to that pixel. To train the NVIDIA semantic segmentation model, the Cityscapes dataset was adopted [42]. The dataset contains over 5000 high-resolution images of urban scenes with detailed annotations of objects and their boundaries. The model was trained to minimize a loss function that measures the difference between the predicted segmentation map and the ground truth segmentation map. The loss function used was the cross-entropy loss, which is commonly used in classification tasks.

In [43], the hierarchical multi-scale attention for semantic segmentation (HMSA) was developed as a modified version of the original NVIDIA semantic segmentation model [41]. The HMSA model introduces additional attention mechanisms and feature aggregation techniques to improve the accuracy of the segmentation. The hierarchical attention mechanism in the HMSA model is composed of three levels of attention modules: a global attention module, a local attention module, and a detail attention module. The global attention module is responsible for capturing the high-level context information of the input image, whereas the local attention module focuses on capturing the mid-level details. The detail attention module is designed to capture the fine-grained details of the input image. Each attention module is implemented as a convolutional neural network (CNN) that takes the feature maps of the previous layer as input and outputs a set of attention maps. The attention maps are used to weigh the feature maps in the previous layer, producing an attention-weighted feature map that is used as input to the next layer of the network. The attention maps are learned via a training process that minimizes the loss function that measures the difference between the predicted segmentation and the ground truth segmentation. In addition to the attention modules, the HMSA model also includes a feature aggregation module that combines features from multiple scales to improve the accuracy of the segmentation. The feature aggregation module is designed to take advantage of the complementary information provided by the features at different scales. Specifically, the feature aggregation module combines the attention-weighted feature maps from each scale using a weighted sum, where the weights are learned via a training process. To further improve the accuracy of the segmentation, the HMSA model uses a multi-scale testing strategy during inference. This strategy involves resizing the input image to multiple scales and passing each resized image through the network to obtain a set of segmentation maps at different scales. The final segmentation map is obtained by upsampling and averaging the segmentation maps obtained at each scale.

The hierarchical multi-scale attention for semantic segmentation (HMSA) model was evaluated using several benchmark datasets, including Cityscapes and PASCAL VOC, and achieved state-of-the-art performance on these datasets. The HMSA model outperformed other semantic segmentation models that do not use attention mechanisms or feature aggregation techniques.

In particular, on the Cityscapes dataset, the HMSA model achieved a mean intersection over union (mIoU) score of 82.2%, outperforming previous state-of-the-art models such as the pyramid scene parsing network (PSPNet) [44] and DeepLabv3+ [45], which achieved mIoU scores of 81.2% and 80.3%, respectively. On the PASCAL VOC dataset, the HMSA model achieved a mIoU score of 88.5%, outperforming previous state-of-the-art models such as DeepLabv3+ and RefineNet, which achieved mIoU scores of 85.7% and 83.4%, respectively.

These results demonstrate the effectiveness of the attention mechanisms and feature aggregation techniques used in the HMSA model, and its ability to outperform previous state-of-the-art semantic segmentation models on benchmark datasets.

**Figure 1:**
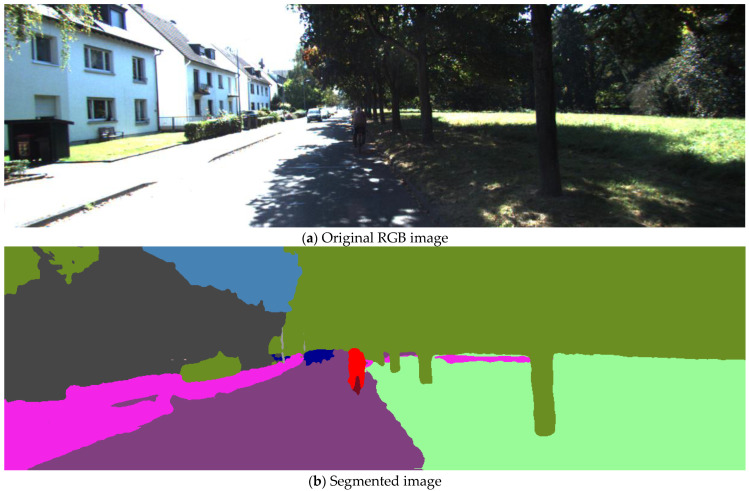
A sample semantically segmented image using NVIDIA semantic segmentation model (KITTI, D-28, frame 1644).

Figure 1 presents a sample of the semantic segmentation results using the NVIDIA semantic segmentation model on the KITTI dataset, D-28. The figure depicts one frame of the dataset, frame 1644.

After completion of the semantic segmentation, the LiDAR points are projected into the image plane. Any LiDAR point that lies on a moving object is neglected. The remaining LiDAR points are used for depth estimation, and thus scale correction.

### 2.3. Proposed Integrated Navigation System

#### 2.3.1. Coordinate Transformations

**Figure 2:**
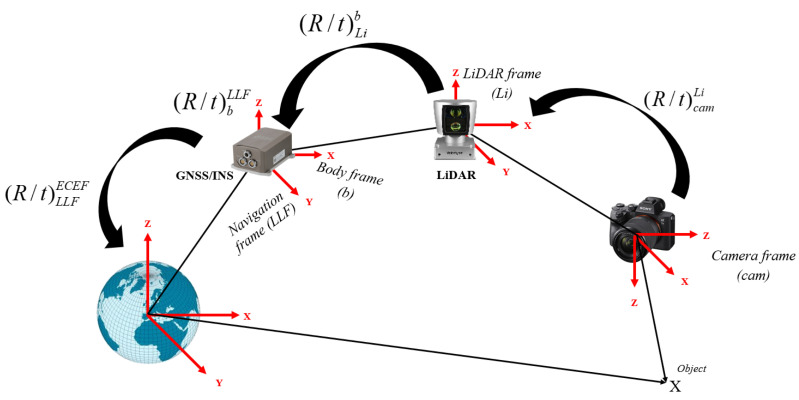
Graphical illustration of camera and LiDAR pose transformation into the world frame (WGS84).

SLAM pose estimations are calculated with respect to the reference frame of the sensor. That is, in LiDAR SLAM, all poses are referenced to the local frame of the first point cloud, whereas in Visual SLAM, they are referenced to the first camera frame. Therefore, all coordinates are transformed into the WGS84 reference, which is essential for subsequent Kalman filtering. The sequence of transformation is graphically shown in Figure 2.

The position and rotation transformations can be performed via a homogenous transformation using 4 × 4 transformation matrices. Let $P_{i}^{cam}$ and $P_{i}^{Li}$ denote the coordinates of a point captured in the local frame of the camera and the LiDAR, respectively. Let $P_{i}^{ecef}$ denote the same point expressed in the WGS84 reference frame. The sequence of homogenous transformations is presented by Equations (1)–(4).

(1) $$P_{i}^{ecef}={\left(R/t\right)}_{l}^{ecef}{\left(R/t\right)}_{b}^{l}{\left(R/t\right)}_{Li}^{b}{\left(R/t\right)}_{cam}^{Li}P_{i}^{cam}$$

(2) $$P_{i}^{ecef}={\left(R/t\right)}_{l}^{ecef}{\left(R/t\right)}_{b}^{l}{\left(R/t\right)}_{Li}^{b}P_{i}^{Li}$$

(3) $$R_{cam}^{l}=R_{b}^{l}R_{Li}^{b}R_{cam}^{Li}$$

(4) $$R_{Li}^{l}=R_{b}^{l}R_{Li}^{b}$$

where ${\left(R/t\right)}_{subscript-origin}^{superscript-destination}$ symbolizes the 4 × 4 homogeneous transformation matrix from the original frame to the destination frame (i.e., ${\left(R/t\right)}_{frame-1}^{frame-2}$: the transformation that transforms coordinates from frame 1 to frame 2; and the frame abbreviations are defined as cam: camera frame, *Li*: LiDAR frame, *b*: body frame, *l*: local-level frame, and *ecef*: WGS84 reference frame.

#### 2.3.2. INS/LIMO Integration

**Figure 3:**
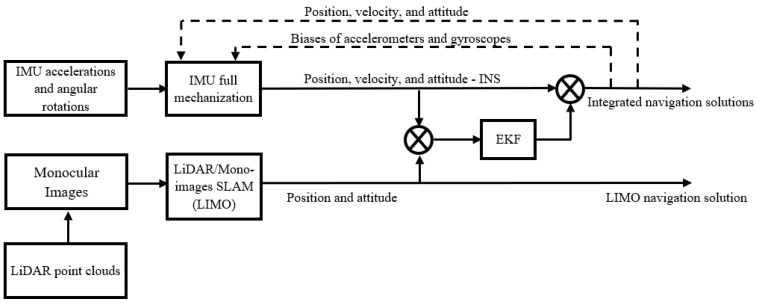
A block diagram of the INS/LIMO LC integration.

The proposed integrated system features a loosely-coupled (LC) integration between the INS and the LIMO, as shown by the block diagram in Figure 3, using an extended Kalman filter (EKF). The raw IMU measurements (i.e., accelerations and angular rotations) are fed into a full IMU mechanization, as described in [30,46], which yields the position, velocity, and attitude of the INS. Meanwhile, both monocular camera images and LiDAR point clouds are fed into the LIMO algorithm to produce the position and attitude estimations. These are used as measurement updates in the update stage of the EKF, which results in the final integrated navigation solution. Finally, the estimated errors are fed back into the full mechanization in a closed-loop error scheme.

The EKF mathematical and stochastic models (i.e., the system model, the design matrix (H), and the system dynamic matrix (F)) are the same as our work in [30]. The system noise covariance matrix (Q) is a diagonal matrix, which includes the noise variance of each parameter in the system state. The state vector and measurement update vector are given by Equations (5) and (6), respectively.

(5) $$\delta x={\left[\begin{array}{ccccc} \delta r & \delta v & \delta \varepsilon & \delta b_{a} & \delta b_{g} \end{array}\right]}^{T}$$

(6) $$\delta Z_{k}={\left[\begin{array}{cc} \delta r & \delta \varepsilon \end{array}\right]}^{T}={\left[\begin{array}{cccccc} \varphi_{NS-LIMO} & \lambda_{NS-LIMO} & h_{NS-LIMO} & p_{NS-LIMO} & r_{NS-LIMO} & y_{INS-LIMO} \end{array}\right]}^{T}$$

where $\delta r={\left[\begin{array}{ccc} \delta \phi & \delta \lambda & \delta h \end{array}\right]}^{T}$ is the position error vector; $\delta v={\left[\begin{array}{ccc} \delta v_{e} & \delta v_{n} & \delta v_{u} \end{array}\right]}^{T}$ is the velocity error vector; $\delta \varepsilon ={\left[\begin{array}{ccc} \delta p & \delta r & \delta y \end{array}\right]}^{T}$ is the attitude angles’ error vector; $\delta b_{a}={\left[\begin{array}{ccc} \delta b_{ax} & \delta b_{ay} & \delta b_{az} \end{array}\right]}^{T}$ is the accelerometer bias error vector; $\delta b_{g}={\left[\begin{array}{ccc} \delta b_{gx} & \delta b_{gy} & \delta b_{gz} \end{array}\right]}^{T}$ is the gyroscope bias error vector; $\phi_{INS-LIMO}$, $\lambda_{INS-LIMO}$, $h_{INS-LIMO}$ are the measurement errors of the geodetic latitude, longitude, and height; and $p_{INS-LIMO}$, $r_{INS-LIMO}$, $y_{INS-LIMO}$ are the measurement errors of the pitch, roll, and yaw angles.

The covariance matrix of the measurements $R_{k-LIMO}$ is assumed to be diagonal and contains the variances of the position and attitude angles received from the LIMO estimations, which are depicted by Equation (7).

(7) $$R_{k-LIMO}=diag\left\{\begin{array}{cccccc} \sigma_{\phi_{LIMO}}^{2} & \sigma_{\lambda_{LIMO}}^{2} & \sigma_{h_{LIMO}}^{2} & \sigma_{p_{LIMO}}^{2} & \sigma_{r_{LIMO}}^{2} & \sigma_{y_{LIMO}}^{2} \end{array}\right\}$$

where $\sigma_{\varphi_{LIMO}}^{2}$, $\sigma_{\lambda_{LIMO}}^{2}$, $\sigma_{h_{LIMO}}^{2}$ are the variances of the LIMO’s position estimations; and $\sigma_{p_{Limo}}^{2}$, $\sigma_{r_{Limo}}^{2}$, $\sigma_{y_{Limo}}^{2}$ are the variances of the attitude estimations of the LIMO algorithm.

#### 2.3.3. INS/LIMO/LiDAR Integration

**Figure 4:**
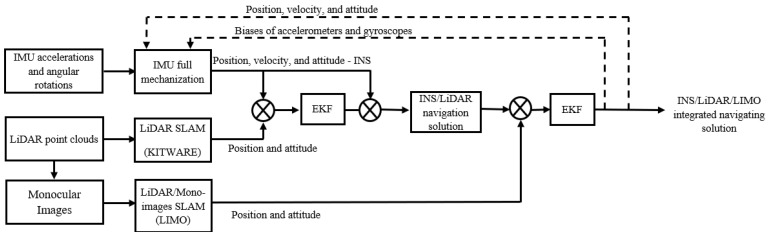
A block diagram of the INS/LIMO/LiDAR LC integration.

As a continuation of our work in [30], we investigated the integration of the INS, LIMO, and LiDAR SLAM as opposed to our proposed INS/LIMO system described in Section 2.3.2. The INS/LIMO/LIDAR integration features the use of a multi-update EKF, as shown by the block diagram in Figure 4. The first update stage of the EKF fuses the INS position and attitude with the LiDAR SLAM pose estimations. The result of the first update stage is INS/LiDAR pose estimations, which are subsequently fused with the LIMO pose estimations in the second update stage to produce the final INS/LIMO/LiDAR integrated navigation model. Finally, the updated errors are fed back into the mechanization to form a closed-loop error scheme.

The state vector is the same as expressed by Equation (5). The first and second update stages are expressed by Equations (8) and (9), respectively.

(8) $$\delta Z_{k1}={\left[\begin{array}{cc} \delta r_{1} & \delta \varepsilon_{1} \end{array}\right]}^{T}={\left[\begin{array}{cccccc} \varphi_{INS-Li} & \lambda_{INS-Li} & h_{INS-Li} & p_{INS-Li} & r_{INS-Li} & y_{INS-Li} \end{array}\right]}^{T}$$

(9) $$\delta Z_{k2}={\left[\begin{array}{cc} \delta r_{2} & \delta \varepsilon_{2} \end{array}\right]}^{T}={\left[\begin{array}{cccccc} \varphi_{NS/Li-LIMO} & \lambda_{NS/Li-LIMO} & h_{INS/Li-LIMO} & p_{INS/Li-LIMO} & r_{INS/Li-LIMO} & y_{INS/Li-LIMO} \end{array}\right]}^{T}$$

where $\delta r_{1}$ and $\delta r_{2}$ are the position error vectors in the first and second update stages, respectively; $\delta \varepsilon_{1}$ and $\delta \varepsilon_{2}$ are the attitude error vectors in both update stages; and $\delta Z_{k1}$ and $\delta Z_{k2}$ are the measurement update vectors. The covariance matrices of the measurement updates are $R_{k}$ and $R_{k-LIMO}$, as shown by Equation (7) and in [30], respectively.

## 3. Data Sources and Driving Scenarios

### 3.1. Scenario 1: Low to Moderate Traffic Environment

In this research, the first scenario involves using the raw Karlsruhe Institute of Technology and Toyota Technological Institute (KITTI, Karlsruhe, Germany) dataset [35], which features driving within low to medium traffic conditions. In addition, it is widely used as a benchmark dataset, which is accessible online via [36]. Researchers worldwide post their results and models on the KITTI odometry benchmark [16]. The sensors used in the KTTTI ground vehicle are an integrated GNSS/IMU unit (OXTS RT3003), a 360-degree rotating mechanical lidar with 64 beams (Velodyne HDL-64E), and two Sony stereo pairs that collect coloured and greyscale images. The dataset includes the estimates of all the system’s intrinsic and extrinsic calibration parameters. The KITTI dataset can be broken down into two main categories, namely, the raw dataset and the odometry dataset. The latter is derived from the raw dataset; however, it does not include IMU raw data measurements (i.e., accelerations and angular rotations). Therefore, the raw dataset is the one considered in this research in order to benefit from the IMU raw measurements, which also contributes as an essential part of the proposed navigation models.

In this study, the KITTI dataset is used to formulate two case studies. The first case study proposes an integrated INS/LIMO navigation model, as presented in Section 4.1—Case Study 1. Meanwhile, the second case study is an extension to our previous work in [30], which is presented in Section 4.3—Case Study 3. This showcases another integrated navigation system (INS/LIMO/LiDAR SLAM) that achieves the concept of sensor redundancy. Table 1 provides a summary and some descriptive trajectory statistics for the raw KITTI drive considered in the aforementioned case studies.

### 3.2. Scenario 2: High Traffic Environment

The second scenario considers driving in high-traffic environments, for which the Leddar PixSet dataset was used [47]. In 2020, this dataset was created by LeddarTech, a company specializing in lidar technology. The data collection platform used by LeddarTech is a ground vehicle (Toyota RAV 4). All perception sensors were mounted on the front bumper of the vehicle, as shown in [47]. The data collection sensors included a solid-state LiDAR (Leddar PIXELL sensor), a mechanical LiDAR (Ouster64), three cameras (FLIR), a 180-deg panorama camera (FLIR), a radar (TI AWR1843 mmWave radar), an integrated GNSS/IMU unit, and a PEAKCAN (Toyota RAV4 CAN bus). The system’s extrinsic and intrinsic calibration parameters are provided for all datasets. In addition, LeddarTech provided researchers with an online Python API to perform a number of analysis tasks on the datasets (i.e., data synchronization and colourizing LiDAR point clouds) [48]. It is highly recommended by the company to use its online API when using its datasets. It is worth mentioning that the datasets were collected in a variety of weather and lighting conditions, and a variety of challenging driving scenarios (occlusions and crowded intersections).

As a result, we considered this dataset, especially the datasets with high traffic, to develop and test the performance of our proposed integrated INS/LIMO navigation system, as presented in Section 4.2 of this paper—Case Study 2. Thse data are recent in the field and the onboard sensor configuration of the data collection platform (front bumper) is different from the KITTI dataset (vehicle top). In addition, the sensors are also of different makes. The drives considered from the Leddar PixSet datasets, along with some descriptive information, are shown in Table 2.

## 4. Analysis and Results

### 4.1. Case Study 1: INS/LIMO Integration—KITTI Dataset

**Figure 5:**
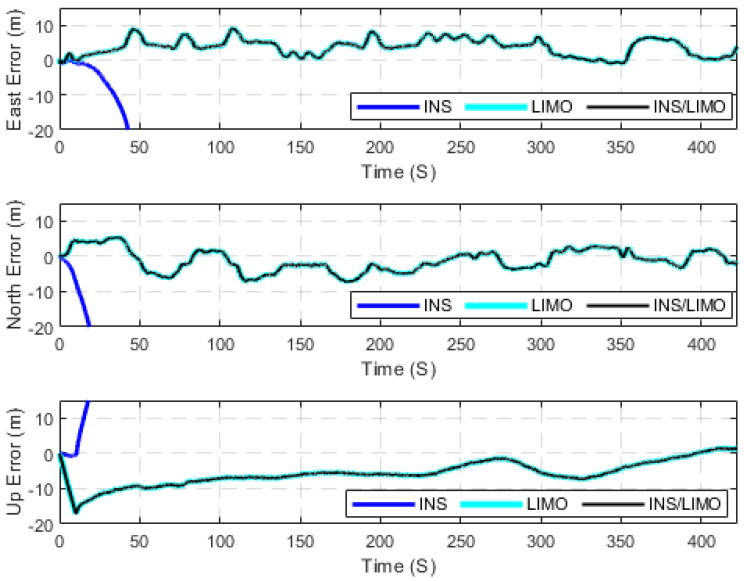
Position errors (ENU), D-28.

**Figure 6:**
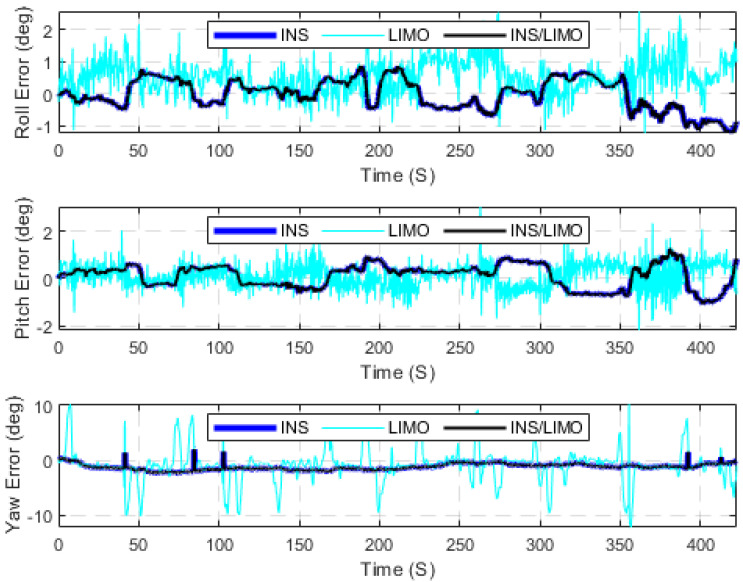
Errors of attitude angles (roll, pitch, and yaw), D-28.

The first case study presents several raw KITTI drives used to test the developed INS/LIMO integrated navigation system. The first dataset 2011_09_30_drive_0028_sync, labelled as D-28, was considered, for which the ground truth is the integrated GNSS/INS solution provided by the OXTS unit operating in the RTK mode. It is worth mentioning that this drive corresponds to sequence number 8 of the KITTI odometry dataset. Consequently, the frames used from raw D-28 are frames 1100 to 5170, which lasted for approximately 408 s, a travelled distance of 3312.20 m, and an average speed of around 28 km/h. Figure 5 presents the errors in the navigation frame (the ENU reference frame). The figure presents three navigation solutions, namely, the INS solution, the LIMO solution, and the INS/LIMO solution. The INS solution drifts significantly in the east, north, and upward directions, which is expected behaviour and echoes our previous work in [30]. Conversely, the LIMO positioning solution is significantly more accurate. As a result, the final INS/LIMO solution followed that of LIMO by tuning the covariance matrix of the system noise (Q) and the covariance of the measurement updates (R). Figure 6 shows the attitude results (roll, pitch, and yaw angles) for the three navigation solutions. In contrast to the positioning results, it is noticeable that the INS produces slightly more accurate attitude results than LIMO as quantified in Table 3. This behaviour also echoes our previous work in [30], where the accuracy of the INS attitude was justified.

However, the INS attitude solution seems to be more stable and significantly less noisy than LIMO. This promotes the INS attitude estimations to be preferable, and thereby the INS/LIMO followed the INS solution by tuning the Q and R matrices. Table 3 presents the errors statistic for both the position and attitude of all navigation solutions. The RMSE values were calculated using Equation (10).

(10) $$\mathrm{RMSE}=\sqrt{\frac{\sum_{i=1}^{n}(x_{i}-\overset{\wedge }{x_{i}})}{n}}$$

where i is an index for each data point, n is the total number of points, and $x_{i}$ and ${\overset{\wedge }{x}}_{i}$ are the estimated value and the ground truth value for each data point, respectively.

It is noticeable from the table that the error statistics of the final integrated INS/LIMO solution are almost identical to the position estimates of the LIMO, albeit the attitude of the INS, which coincides with EKF tuning as mentioned earlier.

**Figure 7:**
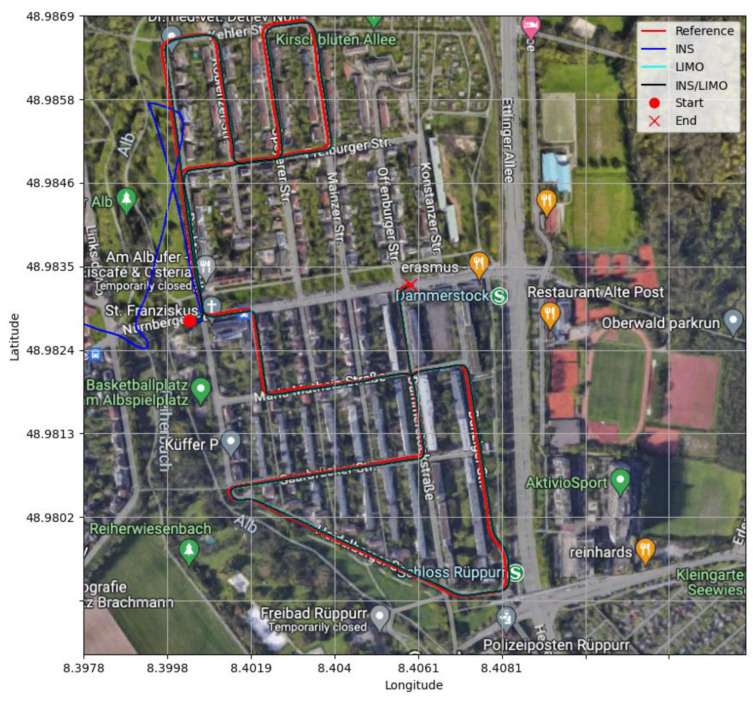
Comparison of trajectories, D-28.

All trajectories of D-28 are visualized and shown in Figure 7, where the ground truth trajectory is compared with the INS, LIMO, and INS/LIMO trajectories.

**Figure 8:**
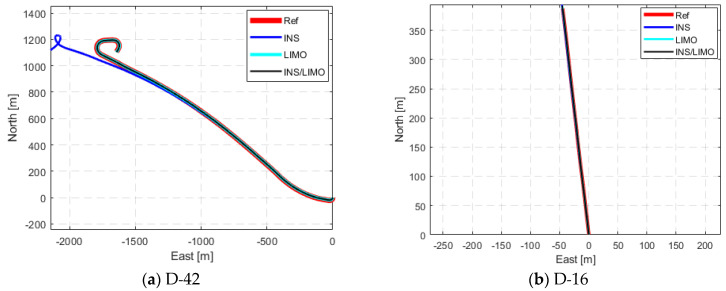

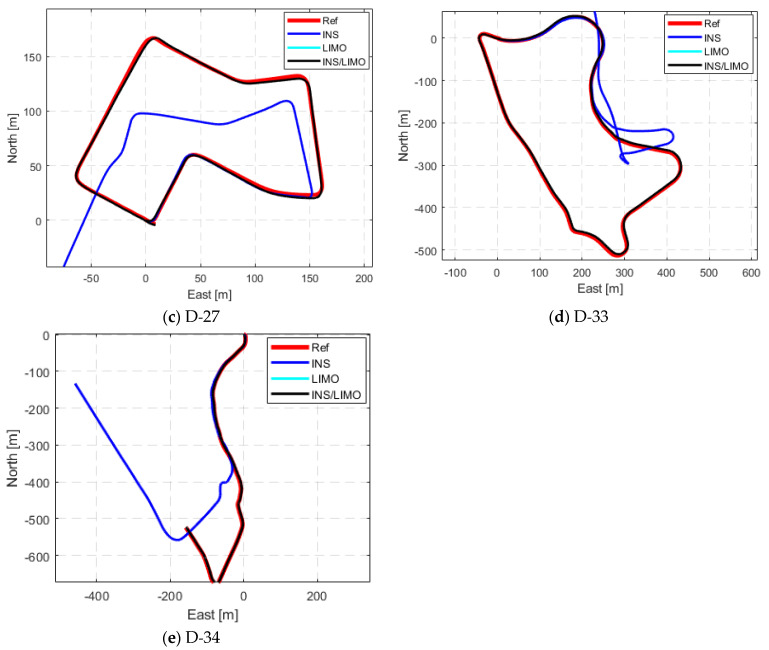
Comparison of trajectories of KITTI drives in the ENU frame.

The same analysis was conducted for drives D-42, D-16, D-27, D-33, and D-34. The drives yielded the same trends for position and attitude. This showcases that the proposed integrated INS/LIMO navigation system is robust and accommodates different driving scenarios in both rural and urban environments. Figure 8 illustrates the comparison of the ground truth trajectory versus all navigation solutions, visualized in the ENU reference frame.

**Figure 9:**
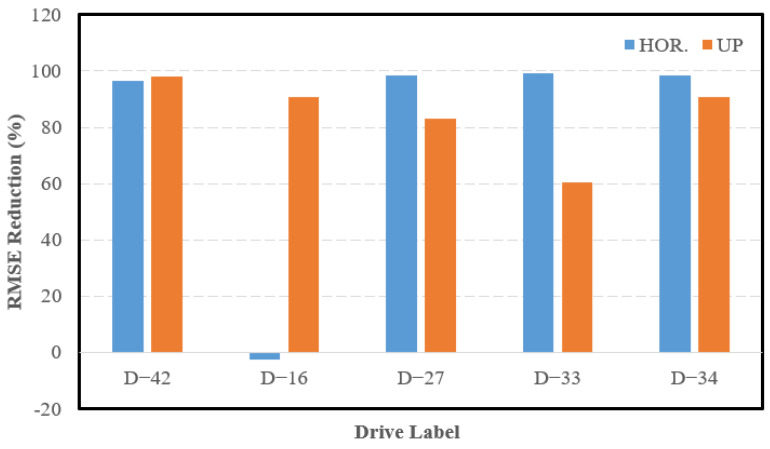
Reduction in the INS RMSE, KITTI drives.

Figure 9 presents the error statistics for the aforementioned drives in the form of a reduction in the RMSE between the INS/LIMO navigation system and the INS solution. Table 4 presents the values of the RMSE in the east, north, horizontal, and upward directions.

The drastic reductions in the horizontal and upward directions for all drives are evident. The only exception is D-16 in the horizontal direction, where the INS slightly outperformed the integrated system solution. The reason for this is that D-16 is very short (roughly 400 m in 28 s). During this short time, the INS solution did not drift significantly. This stability over short periods of time is expected from high-quality IMUs, such as the one used in the KITTI data collection platform. This echoes many similar short drives shown in [30].

### 4.2. Case Study 2: INS/LIMO Integration—LeddarTech PixSet Dataset

**Figure 10:**
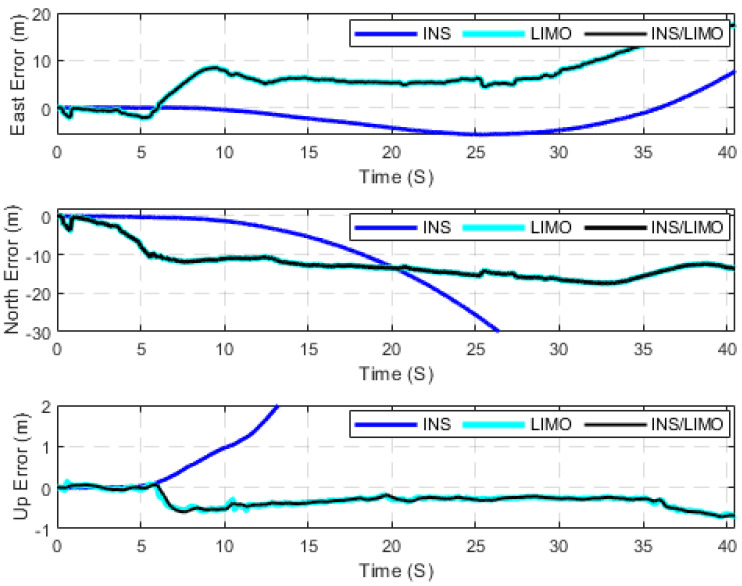
Position errors (ENU), D-1040.

**Figure 11:**
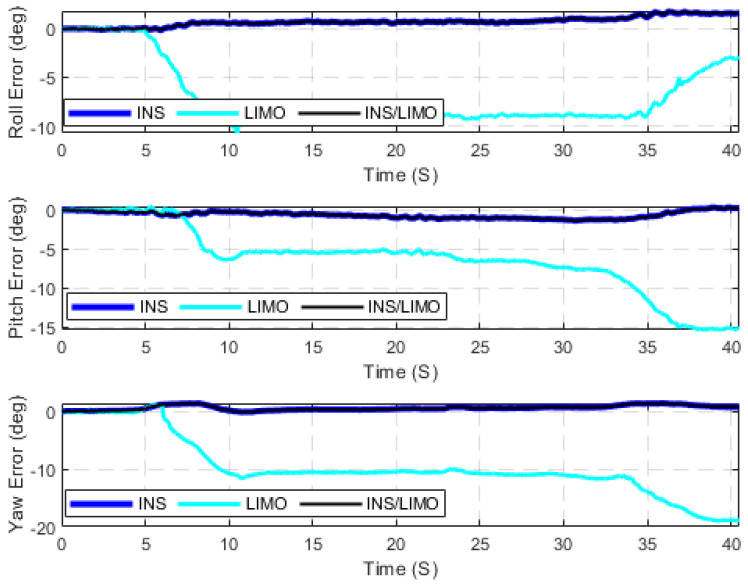
The errors of attitude angles (roll, pitch, and yaw), D-1040.

The second case study adopts the Leddar PixSet dataset as a source for high-traffic driving environments. Therefore, it was considered to further test the developed integrated INS/LIMO navigation system. The first dataset used is 20200721_165008_part39_640_1040, labelled as D-1040. This is an approximately 170 m drive that lasted for 40 s, yielding an average speed of 15 km/h. The ground truth for all drives of the Leddar PixSet dataset is provided by the integrated solution of the GNSS/INS unit (SBG EKINOX) operating in the RTK mode. Figure 10 and Figure 11 present the position and attitude errors of all navigation solutions, respectively. The statistical characteristics of these errors are quantified in Table 5.

In contrast to the first case study (the KITTI dataset), Figure 10 shows that the performance of the LIMO is not quite as accurate as the first case study. However, it still provides a better solution than the INS position estimations. As a result, the integrated navigation solution continues to follow the LIMO solution. In regard to attitude estimations, it can be seen in Figure 11 that INS provides more accurate results for the roll, pitch, and yaw angles, which are similar to those of the first case study. However, it is worth mentioning that the only difference in the comparison to the first case study is the degradation in the quality of LIMO attitude estimations, as shown in Table 4. The reason for such degradation is because of the onboard sensors that are mounted on the front bumper of the car, which narrows the field view of the sensors (both camera and LiDAR) in comparison to the case of the KITTI dataset (car-top mount). This, in turn, makes the entire system sensitive to scene occlusion while driving in high-traffic environments.

**Figure 12:**
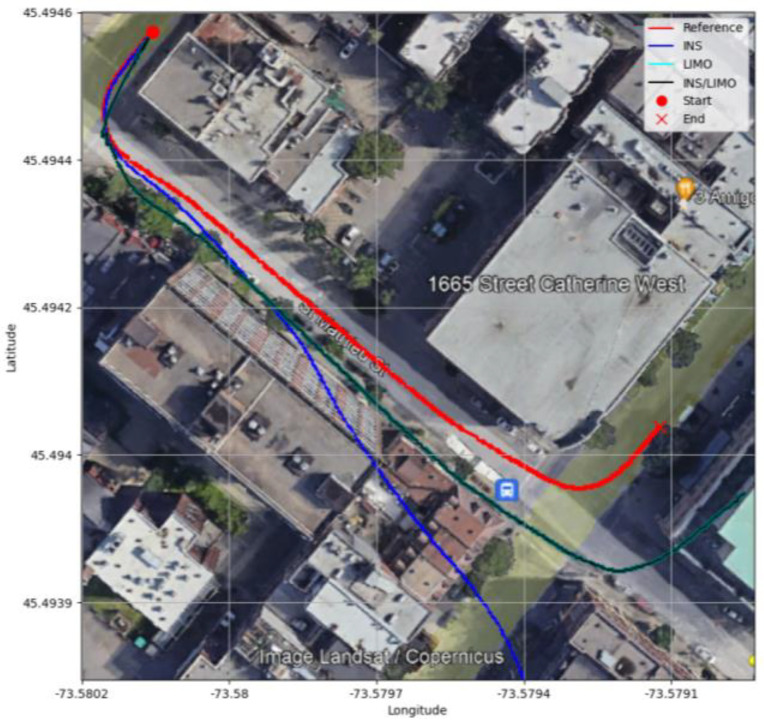
Comparison of trajectories, D-1040.

Figure 12 illustrates a comparison between the ground truth trajectories and all other navigation solutions in the WGS84 reference frame.

**Figure 13:**
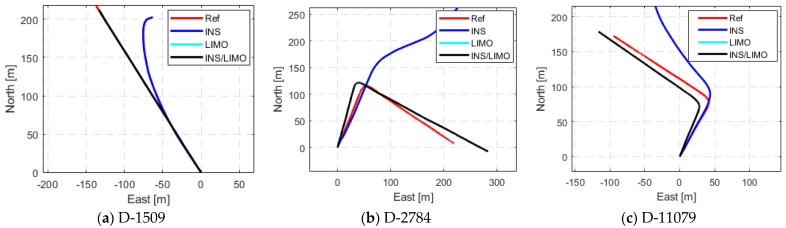
Comparison of trajectories in the ENU frame, Leddar PixSet drives D-1509, D-2784, and D-11079.

**Figure 14:**
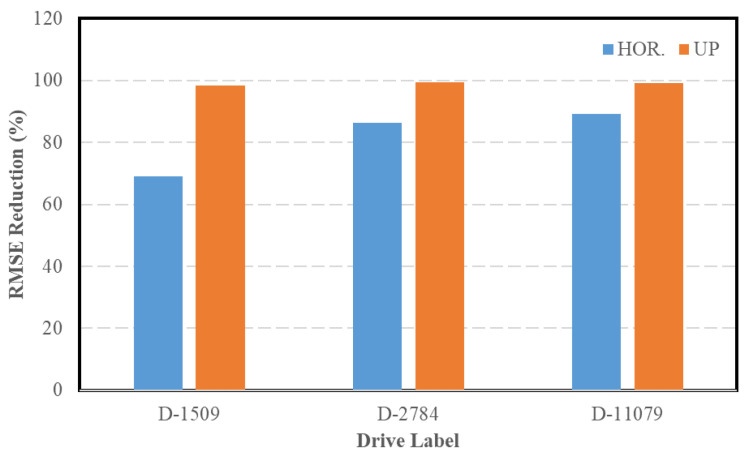
Reduction in the INS RMSE, Leddar PixSet drives.

The integrated navigation system was tested for the remaining drive of the Leddar PixSet dataset. Figure 13 presents a comparison between the trajectories of each of the remaining drives in the ENU reference frame. The reductions in the position RMSE (INS/LIMO vs. INS) in the horizontal and vertical directions are shown in Figure 14, which are quantified in Table 6.

It is important to mention that the same trends of the integrated INS/LIMO system continue to occur among all drives. However, in Figure 13, D-1509 shows that LIMO produces more accurate positioning results in comparison to the other drives. Upon investigation, it is found that D-1509 is a straight driving segment with no turns, with minimal scene occlusions, which is not the case in the remaining drives.

### 4.3. Case Study 3: INS/LIMO/LiDAR Integration—KITTI Dataset

The third and final case study is a continuation of our work in [30], where the integrated INS/LIMO/LiDAR navigation system described in Section 2.3.3 is tested using D-28. The main reason that the KITTI dataset was chosen for this case study is that it is a benchmark dataset that allows for comparison with state-of-the-art models. Furthermore, we used the KITTI dataset in [30], and therefore, we continue to use it in this study to expand our previously proposed INS/LiDAR SLAM navigation model.

**Figure 15:**
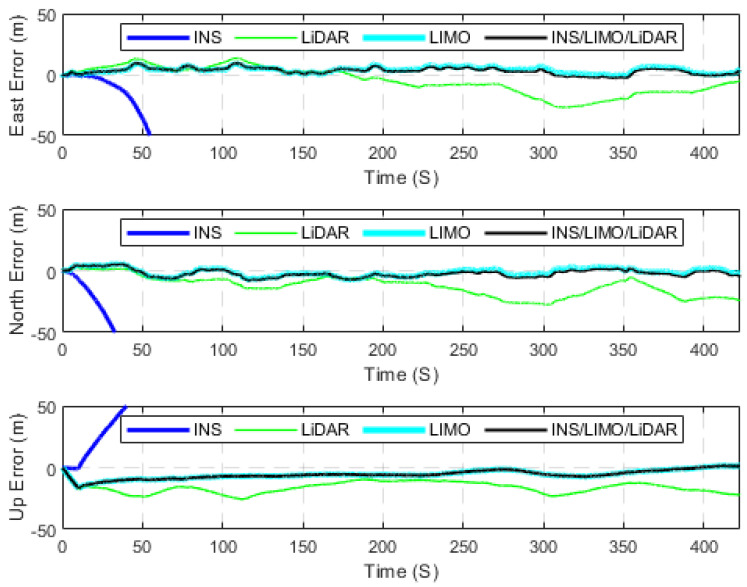
Position errors (ENU), D-28 (INS/LIMO/LiDAR).

**Figure 16:**
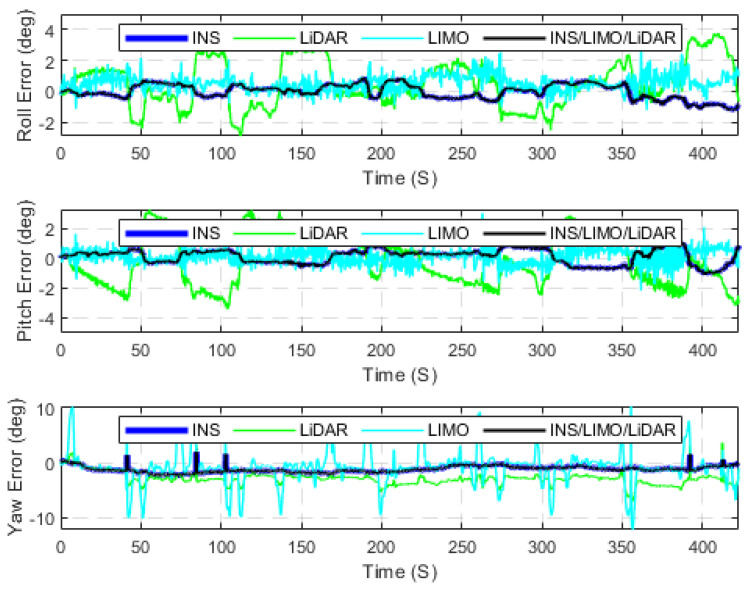
The errors of attitude angles (roll, pitch, and yaw), D-28 (INS/LIMO/LiDAR).

**Figure 17:**
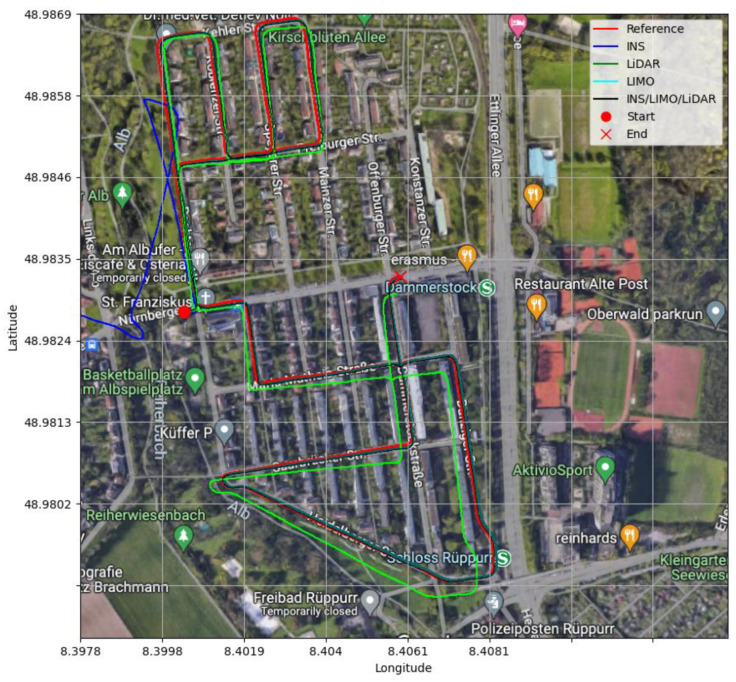
Comparison of trajectories, D-28 (INS/LIMO/LiDAR).

The position and attitude errors are presented in Figure 15 and Figure 16, respectively, whereas Table 7 provides the statistics of these errors. It is noticeable from Table 7 that the INS/LIMO/LIDAR system offers improved accuracy in comparison with LiDAR. The reduction in the RMSE is approximately 70% and 60% in the horizontal and upward directions, respectively. Finally, Figure 17 graphically compares the INS, LIMO, LiDAR SLAM, and INS/LIMO/LiDAR navigation solutions in the WGS84 reference frame.

### 4.4. Comparison between the INS/LIMO and INS/LIMO/LiDAR Integration

It is evident from Figure 15 and Figure 16 that the final integrated navigation system follows the LIMO position estimations and the INS attitude estimations. This means that the performance of the LIMO is superior to the LiDAR SLAM, as solidified by Table 5. As a result, we can conclude that the use of our proposed INS/LIMO integrated system is the best system, given that no sensor failure occurs. In other words, the inclusion of the LiDAR SLAM in the integrated navigation system is redundant. This redundancy enhances the robustness of the navigation system against any potential malfunctioning of the onboard sensors. Therefore, in the case of INS/LiDAR/LIMO integration, the use of the LiDAR as a redundant onboard sensor will prove to be important if the monocular camera fails for any known or unknown reason. Consequently, the navigation system will continue to operate and generate reliable pose estimates up to the accuracy of LiDAR SLAM.

### 4.5. Comparison with State-of-the-Art Models

In order to assess the quality of our proposed navigation model, it is imperative to compare our work with state-of-the-art models. Taking this into account, we used the KITTI dataset in the first and third case studies in this research as a benchmark dataset. In the first case study, our proposed INS/LIMO navigation system provides better attitude estimations in comparison to the sole use of LIMO. In addition, the system positioning estimations rely on LIMO, which outperformed a number of algorithms on the KITTI odometry benchmark [16], such as ORB-SLAM [26,29] and Stereo LSD-SLAM [27].

Regarding the additional integration of INS/LIMO/LiDAR presented in the third case study, we extensively compared the LiDAR SLAM algorithm we used in [30] with leading state-of-the-art LiDAR models [18,20,50]. Our model outperformed the previous models using the KITTI dataset.

## 5. Conclusions

In this research, an integrated INS/Visual SLAM (LIMO) navigation system was proposed. The system featured a loosely-coupled integration of the INS and the monocular camera SLAM pose estimations using an EKF. In addition, the system was tested on two datasets, namely, the KITTI dataset and the Leddar PixSet dataset, which covered various driving environments in terms of the nature of the environment (i.e., rural and urban drives) and traffic intensity (i.e., low to moderate traffic and high traffic). The performance of the integrated system resulted in an average reduction of 80% and 92% in the RMSE in the horizontal and upward directions, respectively. In addition, the proposed navigation system was tested against another integration scheme that fuses the measurement of the IMU, a monocular camera, and a LiDAR sensor. The results showed a reduction of 99% and 97% in the RMSE in the horizontal and upward directions, respectively. In addition, the results also confirmed that the inclusion of LiDAR did not affect the final accuracy of the navigation system, albeit adding sensor redundancy to the system, which can be beneficial in the case of a camera malfunctioning. Finally, our proposed navigation system was compared with state-of-the-art models.

## Figures and Tables

**Table 1:** Trajectory information for the considered KITTI drives [36].

Drive Label	Drive Number	Length (m)	Time (s)	Average Speed (km/h)	No. of Frames
D-27	2011_09_30_drive_0027_sync	692.47	114.85	21.71	1106
D-28	2011_09_30_drive_0028_sync	3312.20	407.80	28.17	4078
D-33	2011_09_30_drive_0033_sync	1709.57	165.31	37.23	1594
D-34	2011_09_30_drive_0034_sync	920.52	126.88	26.12	1224
D-16	2011_09_30_drive_0016_sync	404.71	28.84	50.52	278
D-42	2011_10_03_drive_0042_sync	2591.80	121.19	76.99	1170

**Table 2:** Trajectory information for the considered Leddar PixSet drives [49].

Drive Label	Drive Number	Length (m)	Time (s)	Average Speed (km/h)	No. of Frames
D-1040	20200721_165008_part39_640_1040	169.29	40.60	15	407
D-1509	20200618_191030_part17_1120_1509	258.19	38.99	23.83	391
D-2784	20200706_144800_part25_2160_2784	329.70	68.30	17.38	684
D-11079	20200706_171559_part27_10588_11079	258.15	50.10	18.55	502

**Table 3:** Position (m) and attitude angles (deg) error metrics—D-28.

	INS			LIMO			INS/LIMO		
	Mean	RMSE	Max	Mean	RMSE	Max	Mean	RMSE	Max
East	−1817.44	2475.40	5675.91	3.74	4.44	9.17	3.74	4.44	9.17
North	−895.41	1036.99	1544.63	−1.22	3.33	7.31	−1.22	3.33	7.31
Horizontal	2092.57	2683.83	5706.95	5.12	5.55	9.47	5.12	5.55	9.47
Upward	262.23	298.13	483.47	5.81	6.61	17.17	5.81	6.61	17.19
Roll	−0.018	0.470	1.171	0.529	0.731	2.563	−0.018	0.474	1.199
Pitch	0.114	0.497	1.238	0.112	0.506	3.026	0.115	0.504	1.278
Yaw	−1.226	1.344	2.348	−0.919	3.144	12.207	−1.248	1.361	2.350

**Table 4:** Positional RMSE (m) values for the KITTI drives.

	D-42		D-16		D-27		D-33		D-34	
	INS	INS/LIMO	INS	INS/LIMO	INS	INS/LIMO	INS	INS/LIMO	INS	INS/LIMO
East	194.48	4.80	0.79	0.40	43.21	1.17	73.09	2.06	114.53	1.58
North	32.71	4.84	3.15	3.30	133.18	1.84	378.77	2.81	136.57	2.38
Horizontal	197.21	6.81	3.25	3.33	140.02	2.18	385.76	3.48	178.24	2.86
Upward	22.21	0.46	1.83	0.17	10.48	1.78	13.62	5.41	12.74	1.16

**Table 5:** Position (m) and attitude angles (deg) error metrics—D-1040.

	INS			LIMO			INS/LIMO		
	Mean	RMSE	Max	Mean	RMSE	Max	Mean	RMSE	Max
East	−1.87	3.44	7.77	6.65	8.20	17.56	6.65	8.20	17.56
North	−25.94	38.66	98.13	−12.27	12.97	17.45	−12.27	12.97	17.45
Horizontal	26.21	38.82	98.43	14.38	15.35	22.24	14.38	15.35	22.24
Upward	5.86	7.59	14.75	0.31	0.35	0.70	0.31	0.35	0.71
Roll	0.725	0.863	1.802	−6.935	7.664	10.717	0.730	0.871	1.822
Pitch	−0.623	0.765	1.362	−6.231	7.544	15.388	−0.629	0.770	1.375
Yaw	0.586	0.715	1.382	−9.687	10.935	18.966	0.584	0.713	1.374

**Table 6:** Positional RMSE (m) values for the Leddar PixSet drives.

	D-1509		D-2784		D-11079	
	INS	INS/LIMO	INS	INS/LIMO	INS	INS/LIMO
East	31.41	5.49	55.68	25.07	16.51	11.96
North	10.66	8.71	184.15	7.11	117.17	4.81
Horizontal	33.17	10.29	192.39	26.06	118.33	12.90
Upward	4.60	0.08	19.31	0.10	10.80	0.09

**Table 7:** Position (m) and attitude angles (deg) error metrics—D-28 (INS/LIMO/LIDAR).

	INS			LIMO			LiDAR			INS/LIMO/LiDAR		
	Mean	RMSE	Max	Mean	RMSE	Max	Mean	RMSE	Max	Mean	RMSE	Max
East	−1817.44	2475.40	5675.91	3.74	4.44	9.17	−5.06	12.10	27.00	3.74	4.44	9.17
North	−895.41	1036.99	1544.63	−1.22	3.33	7.31	−12.32	14.70	27.64	−1.22	3.33	7.31
Horizontal	2092.57	2683.83	5706.95	5.12	5.55	9.47	16.80	19.05	36.51	5.12	5.55	9.47
Upward	262.23	298.13	483.47	5.81	6.61	17.17	16.25	16.84	25.64	5.81	6.61	17.19
Roll	−0.0177	0.4696	1.1714	0.5292	0.7313	2.5635	0.5358	1.5920	3.7675	−0.0177	0.4738	1.1978
Pitch	0.1140	0.4971	1.2378	0.1118	0.5056	3.0260	−0.1281	1.7569	3.4024	0.1150	0.5040	1.2745
Yaw	−1.2263	1.3443	2.3483	−0.9188	3.1443	12.2069	−2.9487	3.1716	7.0003	−1.2612	1.3703	2.3497

## Data Availability

The data used in this study can be found at: http://www.cvlibs.net/datasets/kitti/ (accessed on 10 November 2022) and https://dataset.leddartech.com (accessed on 15 November 2022).
